# Interleukin-4 can play a role in allergic rhinitis patient during treatment with *Zataria multiflora*

**DOI:** 10.1186/s12948-022-00169-w

**Published:** 2022-02-10

**Authors:** Nazila Ariaee, Yaser Yadegari, Mohamad Shabestari, Javad Asili, Maryam Panahi, Jalal Ghorbani, Farahzad Jabbari

**Affiliations:** 1 Allergy Research Center, Mashhad University of Medical Sciences, Mashhad, Iran; 2 Preventive Cardiovascular Care Research Center, Mashhad University of Medical Sciences, Mashhad, Iran; 3 Department of Pharmacognosy, School of Pharmacy, Mashhad University of Medical Sciences, Mashhad, Iran; 4 Department of Emergency Medicine, Faculty of Medicine, Mashhad University of Medical Sciences, Mashhad, Iran

**Keywords:** Allergic rhinitis, Cytokines, IL-4, Shirazi thyme, *Zataria multiflora*

## Abstract

**Background:**

Allergic rhinitis is a widespread disorder across the globe. The Shirazi thyme (*Zataria multiflora*) has been shown to have considerable antioxidant and anti-inflammatory properties. This study assessed the effect of this herbal product on alterations in inflammatory/anti-inflammatory cytokines.

**Method:**

This study was conducted on the bank sample before and after the intervention to measure interleukin-4, interleukin-5, and interferon -γ levels with the ELISA test method in a supernatant taken from the PBMC cell culture from 30 allergic rhinitis patients.

**Results:**

The IL-4 level had no significant difference between the two groups before the treatment. However, it had a significant increase in the case group after the treatment. The IL-5 level was significantly higher in the case group before the treatment. Nevertheless, there were no significant differences between the case and control groups after the treatment. Similarly, no significant differences were observed between the two groups considering IFN-γ before and after the treatment.

**Conclusion:**

Consuming thyme with an increase in anti-inflammatory cytokine IL-4 and a decrease in IL-5 cytokine control inflammation and improvement in allergic rhinitis symptoms.

*Clinical trial details* This clinical trial study was recorded at 22.5.2014 in the Iran Registry of Clinical Trials code: (IRCT2016121823235N6) https://www.irct.ir/trial/19852

## Introduction

Allergic rhinitis is a highly prevalent inflammatory disorder of the nasal mucosa with symptoms including nose itching, rhinorrhea, sneezing, and a congested nose, and disabling effects. Harmful effects of allergic rhinitis on the quality of life and education have been proven. The prevalence of this disorder has been reported to be from 4.3% to 45% in different parts of the world [1]. Allergic rhinitis can be associated with other allergic disorders [2]. Exposure to allergens in atopic hosts leads to production of IgE, and with secondary exposure, early and late allergic responses lead to development of symptoms [3].

On the one hand, the production and increase of specific allergen IgE is a typical laboratory manifestation in atopic allergic rhinitis [4]. Main cytokines interleukin-4 (IL-4), interleukin-5 (IL-5), and interleukin-13 play a critical role in the production of atopic allergic rhinitis [5], and along with IFN-y, cause continuous allergic inflammatory processes [6]. On the other hand, *Zataria multiflora*, commonly called Shirazi thyme, is a local herb found in Iran and other parts of the Middle East. The plant’s extract contains phenolic compounds (thymol and carvacrol) and the main non-phenolic compound, which all have proven antioxidant and anti-inflammatory effects such as irritable bowel syndrome, infectious diseases and bronchitis [7].

Accordingly, we planned to investigate whether this herbal product (ZM extract) is helpful for patients with allergic rhinitis. Our previous results demonstrated an improvement in the allergic rhinitis symptoms after using thyme compared to the placebo group [8]. Therefore, in this study, we aimed to further evaluate the effect of the ZM extract on patients with allergic rhinitis. To this end, the TH-1/TH-2 balance was defined by measuring IL-4, IL-5, IFN-y cytokines in patients with allergic rhinitis before and after treatment with the ZM extract.

## Materials and methods

### Patients

This study was conducted on the sample bank of Ariaee et al.’s study [8]. The Iranian Registry of Clinical Trials approved this investigation (code: IRCT2016121823235N6). The samples included 30 patients suffering from seasonal allergic rhinitis whose disorders were proven by an allergist according to the clinical criteria and the positive skin prick test for common local aeroallergens [9]. After collecting consent forms, the patients were randomly divided into two groups to receive either the thyme product or the placebo, which marked and differentiated by the producer with A and B labels. Group A was defined as the control group, while Group B was determined as the case group. The products distributor provided both groups with A syrup or B syrup using the envelope method. Neither the distributor nor the patients knew about what the label represented. All the patients were equally placed under treatment of a daily dose of 30 ml of thyme syrup in addition to their standard treatment of the disorder (daily consumption of 10 mg of cetirizine and two puffs of Floxinase nasal spray). In this study, samples consisted of 10 cc intravenous blood were taken from patients before and two months after the intervention. The lymphocytes were separated by Ficoll and cultured for 24 h. Then, the upper part of the samples (supernatant) was collected after stimulation with a phytomito-antigen mitogen factor and then was centrifuged and frozen at − 80 °C. Each patient consumed 30 cc of the syrup three times a day (overall, receiving approximately 6 mg of thymol, 0.85 mg of carvacrol, and 7 mg of total phenol daily). The placebo was provided in a precisely identical manner, with no active ingredients and some similar thyme aromatic compounds to make the product.

### Laboratory analysis

Peripheral blood mononuclear cells (PBMCs) were isolated, then activated by phorbol myristate acetate (25 ng/ml) (Sigma-Aldrich, Germany) and ionomycin (1 µg/ml) (Sigma-Aldrich, Sigma, Germany) for 6 h. The supernatant of the activated PBMCs was harvested after 48 h for the cytokine assay and banked. The concentration of IL-4, IL-5, and IFN-γ in the serum samples and culture supernatants was determined using a commercial ELISA kit (eBioscience, Austria) according to the manufacturer’s instructions.

The ZM extract was made from 20% of a hydroalcoholic ZM extract, Giah Essence (Gorgan, Iran) in the form of a syrup containing 37% of a hydroalcoholic Shirazi thyme extract (every 100 cc syrup, including 37% of the extract and 63% of other substances) with an alcohol content of 4%. Every vial contained 120 cc syrup with active ingredients, as previously mentioned [8].

### Statistical analysis

Data was analyzed using the Kolmogorov–Smirnov test and the Lilliefors test in IBM-SPSS v.22.

In case of normal results, student parameter methods were used otherwise the Mann–Whitney-Wilcoxon statistical test was applied. Furthermore, Pearson’s Chi-square test and Fisher’s exact test were used for data analysis. The analysis method was conducted as per protocol. It should be noted that the p-value was considered below 0.05.

## Results

The minimum and maximum age of the participants were 11 and 67 years respectively, with a mean score of 32.23 ± 13.09 years. The average age was 33.86 ± 16.7 in the control group and 30.6 ± 8.39 in the case group. The age distribution in each group was normal, and there was no significant difference between the two groups in terms of age. There was a significant difference between the two groups regarding gender; three men and 12 women were in the control group, while ten men and five women being in the case group.

SNOT22 total scores were compared during the study period considering the pairwise test. Based on the SNOT-22 questionnaire, nose and ear complications, sleep disorders, psychological issues, and daily activities were evaluated before and after intervention. As shown in Table 1, it was observed that the ZM syrup has stronger alleviatory effects than the placebo, considering nose related symptoms and sleep state among the entire parameters in SNOT22.

**Table 1:** The mean score of the patients’ symptoms (placebo and ZM group), before and after the intervention based on the SNOT-22 questionnaire

Symptoms	Groups	Before intervention mean ± SD	After intervention mean ± SD	P value
Nose	ZM	37 ± 3.6	19 ± 1.2	< 0.0001
	Placebo	43 ± 4.4	41 ± 3.4	
Ear	ZM	3.1 ± 0.2	2.9 ± 0.1	0.78
	Placebo	3.1 ± 0.5	2.9 ± 0.3	
Sleep state	ZM	9 ± 0.65	2.5 ± 1.4	< 0.0001
	Placebo	9.2 ± 0.86	8.7 ± 0.8	
Activity	ZM	8.8 ± 3.1	4.1 ± 1.1	0.47
	Placebo	8.1 ± 2.3	4.3 ± 1.5	
Psychology	ZM	7.3 ± 1.6	6.4 ± 3.1	0.9
	Placebo	8.1 ± 1.1	6.9 ± 3.4	

**Fig. 1:**
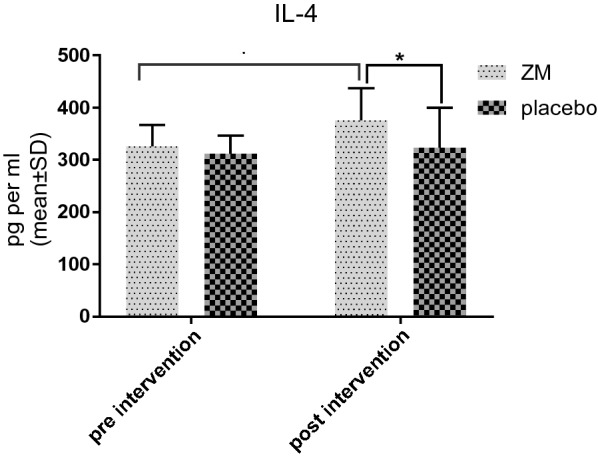
IL-4 level in supernatant of PBMC culture before and after the intervention with ZM syrup

Evaluating cytokine IL-4 level in this intervention represented no significant differences between the two groups before the treatment. However, a significant difference (p value: 0.046) was observed between the two groups regarding IL-4 levels after the treatment; accordingly, IL-4 levels were higher in the case group than in the control group after the treatment. While there was no significant difference before and after the treatment in the control group, IL-4 levels in the case group showed a significant increase following the treatment (Fig. 1).

**Fig. 2:**
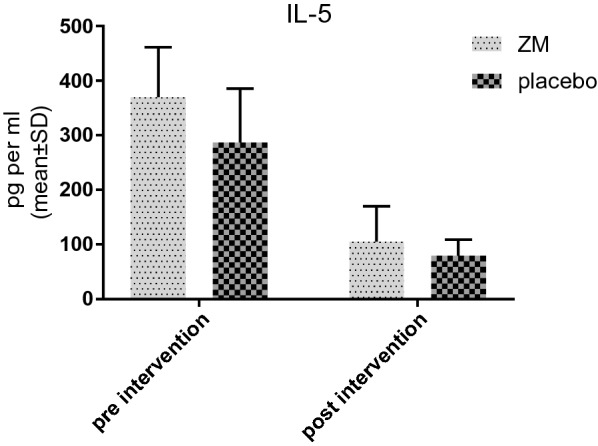
IL-5 level in supernatant of PBMC culture before and after the intervention with ZM syrup

Assessment the amount of IL-5 in the supernatant of PBMCs culture indicted the average IL-5 level was higher in the case group. Moreover, a significant difference between the two groups (ZM and placebo) before the treatment was illustrated. After the intervention, IL-5 levels decreased in both case and control groups. Although the difference was significant, it was a valueless data since it was seen in the both groups. Therefore, there were no significant differences between the two groups regarding IL-5 levels after the treatment (Fig. 2).

**Fig. 3:**
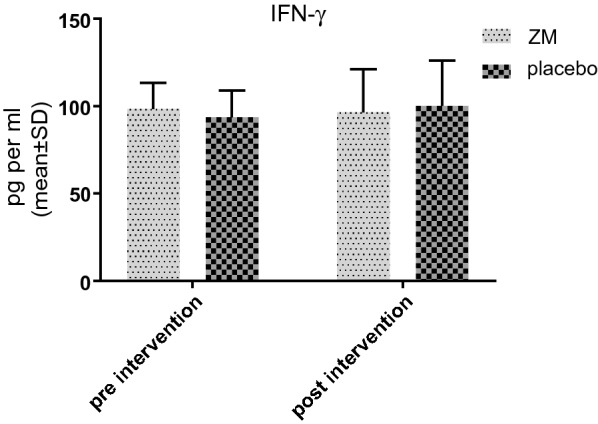
IFN-Ƴ level in supernatant of PBMC culture before and after the intervention with ZM syrup

Prior to the treatment, the concentration of IFN-γ in ZM group was very close to the placebo group without any significant difference. After the treatment, no significant differences were observed in the both groups. After administration of ZM syrup, the amount of this cytokine dropped, though this decrease was the same as placebo group. Hence, it cannot reach statistical difference. The details can be seen in Fig. 3.

## Discussion

After two months of thyme consumption in our case group, the anti-inflammatory IL-4 level in the supernatant cell culture of PBMCs had a substantial decrease compared to the time before the treatment.

Extensive studies have been conducted on allergic rhinitis disorder, its symptoms and various treatments. Naturally, compounds are an important source for clinical proposes [10]. In recent years, studies have been conducted on herbal medicines and their effects. For instance, many beneficial effects have been reported for the Shirazi thyme plant, and a number of them have been proven [11]. As the role of cytokines is highlighted in many studies, it was attempted in the present study to evaluate the role of cytokines [12, 13]. In our previous study, the effects of Shirazi thyme consumption were evaluated on the clinical symptoms and severity of allergic rhinitis according to the SNOT22 questionnaire. We observed that the severity of the clinical symptoms in both case and control groups was improved after the treatment compared to the time prior to the treatment. Nevertheless, the severity of the clinical symptoms in the case group was dramatically greater after the treatment in contrast to the time before the treatment [14].

Studies have shown that the IL-4 cytokine, despite producing IgE that enhances allergy, can also produce IgG4, which is a blocking antibody and plays the role of anti-inflammatory cytokines. Likewise, the IL-5 level, an eosinophil recruitment cytokine, decreased in the supernatant cell culture of PBMCs after the course of treatment compared to the previous time in both groups. Regarding the fact that the level of IL-5 cytokine in the supernatant cell culture of PBMCs within the case group was higher before the start of treatment, the effect of thyme on decreasing its level was more likely than the control group [15]. Therefore, these two findings (the increase in IL-4 and the decrease in IL-5) are consistent with the findings of studies showing the effects of Shirazi thyme consumption on the clinical symptoms. Moreover, the allergic rhinitis severity was evaluated according to the SNOT22 questionnaire. The severity of the clinical symptoms in both groups was better after the treatment in comparison with the before time. Nevertheless, the severity of the clinical symptoms in the case group was dramatically greater by the treatment. Similarly, the effects of the ZM syrup were evaluated on the gene expression rate in PBMC cells in the allergic rhinitis patients. The results presented that the TGF-β gene expression had no change in the control group and a slight decrease in the case group. The IL-10 gene expression also decreased in both control and case groups, although this reduction was more significant in the case group. The IL-17 gene expression decreased in the both groups, while it was significant only in the case group. Further, the FOXP3 gene expression decreased in both control and case groups, though it was more significant in the case group. They illustrated that IL-4 cytokines caused damage to Th17 cells. These cells are producers of IL-17 inflammatory cytokines [16]. Accordingly, an increase in IL-4 in our study confirms with the reduced IL-17 in our previous study [8].

It was also demonstrated that the Thymol extract, the Flavonoid extract and the essence of this herb contained anti-inflammatory effects. In applying the administered dose, there was no significant difference in the anti-inflammatory effects of different fractions of the Shirazi thyme with the standard group receiving dexamethasone [7]. Moreover, it became clear that the prevention of scar formation was another effect of the Shirazi thyme herb, which can be used as an anti-inflammatory drug in the future by extracting its effective substances [17, 18]. Due to the fact that IL-4 is an anti-inflammatory cytokine, by demonstrating the augmentation of IL-4 in patients consuming thyme, our study is probably able to confirm its anti-inflammatory effects.

A study was conducted in 2015 on the effects of the Shirazi thyme and the carvacrol compounds on inflammatory pulmonary changes and oxidative stress in guinea pigs suffering from COPD due to their exposure to cigarette smoke. The effect of this medicine was evaluated on blood cell total count, eosinophils, neutrophils count demonstrated in groups treated with dexamethasone, two high concentrations of the Shirazi thyme, and two concentrations of carvacrol. Changes toward recovery were observed dramatically. Dissimilar results in the control group was significant indicating the protective effects of the Shirazi thyme extract and its active ingredients, including carvacrol [19].

Other investigations on guinea pig groups were conducted to evaluate the effect of the Shirazi thyme on the responsiveness of the trachea in the pathologic lung. Their results illustrated that the groups afflicted with COPD had a significant improvement compared to the control group [20].

Emphysema in the group under treatment with the highest thyme and carvacrol concentrations had improved significantly compared to the group of patients without the treatment. Moreover, an almost equal effect was achieved by the Shirazi thyme compared to the effect of Dexamethasone. Furthermore, the anti-inflammatory effects of this herb were observed in our study. Based on its effect on a number of cytokines, they are the most important mediators of the immune system responses.

In addition, a study was conducted in 2011 on the effects of another herbal product (silymarin) on the treatment of chronic allergic rhinitis. The results clarified that the severity of the clinical symptoms of allergic rhinitis after the treatment with silymarins had a significant improvement, compared to the placebo group. The cytokines’s (IFN-γ, IL-4, and IL-5) serum levels did not significantly differ between the case and control groups after the treatment [21]. Accordingly, the finding of the mentioned study does not match the variations of cytokines in our study. It can be explained by different study durations. Their study took one month, whereas our study lasted for two months. It is notable that a longer duration of medication provided a better effect on the immune system.

Overall, due to the proven effects of the Shirazi thyme herb on the treatment of inflammatory diseases and its availability in Iran, the consumption of this relatively safe herb (according to our study and other studies) is recommended. It is effective for inflammatory disorders, especially allergic rhinitis alongside the other treatments so as to further control patients’ symptoms. Moreover, due to the impact of the Shirazi thyme on the improvement of patients’ clinical symptoms, it is suggested to study the total and specific IgE level, and the number of eosinophils in the nasal mucosa on a significant number of patients. In addition, the clinical symptoms and previous cytokine levels should be observed.

## Conclusion

Since thyme is a popular condiment and cost-effective herb, it would likely be well-accepted by patients. Therefore, it is suggested to be taken in addition to the conventional treatment. It may alleviate via increasing of IL-4 expression level.

## Data Availability

All relevant data are included in the manuscript.
